# Leukocytosis and Enhanced Susceptibility to Endotoxemia but Not Atherosclerosis in Adrenalectomized APOE Knockout Mice

**DOI:** 10.1371/journal.pone.0080441

**Published:** 2013-11-12

**Authors:** Menno Hoekstra, Vanessa Frodermann, Tim van den Aardweg, Ronald J. van der Sluis, Johan Kuiper

**Affiliations:** Division of Biopharmaceutics, Leiden Academic Centre for Drug Research, Leiden, The Netherlands; King’s College London School of Medicine, United Kingdom

## Abstract

Hyperlipidemic apolipoprotein E (APOE) knockout mice show an enhanced level of adrenal-derived anti-inflammatory glucocorticoids. Here we determined in APOE knockout mice the impact of total removal of adrenal function through adrenalectomy (ADX) on two inflammation-associated pathologies, endotoxemia and atherosclerosis. ADX mice exhibited 91% decreased corticosterone levels (P<0.001), leukocytosis (WBC count: 10.0 ± 0.4 x 10E9/L vs 6.5 ± 0.5 x 10E9/L; P<0.001) and an increased spleen weight (P<0.01). FACS analysis on blood leukocytes revealed increased B-lymphocyte numbers (55 ± 2% vs 46 ± 1%; P<0.01). T-cell populations in blood appeared to be more immature (CD62L+: 26 ± 2% vs 19 ± 1% for CD4+ T-cells, P<0.001 and 58 ± 7% vs 47 ± 4% for CD8+ T-cells, P<0.05), which coincided with immature CD4/CD8 double positive thymocyte enrichment. Exposure to lipopolysaccharide failed to increase corticosterone levels in ADX mice and was associated with a 3-fold higher (P<0.05) TNF-alpha response. In contrast, the development of initial fatty streak lesions and progression to advanced collagen-containing atherosclerotic lesions was unaffected. Plasma cholesterol levels were decreased by 35% (P<0.001) in ADX mice. This could be attributed to a decrease in pro-atherogenic very-low-density lipoproteins (VLDL) as a result of a diminished hepatic VLDL secretion rate (-24%; P<0.05). In conclusion, our studies show that adrenalectomy induces leukocytosis and enhances the susceptibility for endotoxemia in APOE knockout mice. The adrenalectomy-associated rise in white blood cells, however, does not alter atherosclerotic lesion development probably due to the parallel decrease in plasma levels of pro-atherogenic lipoproteins.

## Introduction

Apolipoprotein E (APOE) is a multi-functional anti-atherogenic molecule secreted by hepatocytes and, to a lesser extent, bone marrow-derived cells such as macrophages. APOE facilitates the efflux of cholesterol from lipid-laden macrophages [1], lowers cellular lipid oxidation [2], and inhibits the proliferation and migration of smooth muscle cells [3]. However, APOE is best known for its anti-atherogenic effect on the metabolism of lipoproteins. APOE is primarily associated with lipoprotein remnants that are cleared from the blood circulation through binding to heparan sulfate proteoglycans (HSPG) for subsequent uptake by the LDL receptor (LDLR) and LDL receptor-related protein 1 (LRP1) located on hepatocytes [4]. Wild-type mice are not susceptible to atherosclerosis. In contrast, disruption of total body APOE function, i.e. in genetically modified APOE knockout mice, is associated with severe hyperlipidemia and spontaneous development of atherosclerotic lesions already on a regular chow low fat diet without added cholesterol [5]. APOE knockout mice do also exhibit an enhanced susceptibility to endotoxemia [6,7], which supports the concept that the presence of hyperlipidemia renders these mice more prone for (lethal) inflammation.

Glucocorticoids, also known as stress hormones, are a class of steroids that are secreted by cortical cells of the zona fasciculata within the adrenals in response to activation of the hypothalamus-pituitary-adrenal (HPA)-axis. Glucocorticoids represent a class of potent immunosuppressive molecules (reviewed by Baschant and Tuckermann [8]). Glucocorticoids suppress neutrophil rolling, adhesion and transmigration, suppress macrophage activation and dendritic cell maturation and migration, and induce apoptosis of dendritic cells and lymphocytes. Our previous studies in low-density lipoprotein (LDL) receptor knockout mice have suggested that glucocorticoids through their immunosuppressive action protect against the development of atherosclerotic lesions upon feeding an inflammatory cholate-containing high cholesterol/high fat diet that stimulates adrenal steroidogenesis and induces severe hyperlipidemia [9]. Interestingly, findings by Raber et al. [10] and Grootendorst et al. [11] have indicated that APOE knockout mice already when fed a standard low fat chow diet display a higher basal level and action of glucocorticoids. These combined findings suggest that the enhanced glucocorticoid action may represent an inherited protective anti-inflammatory response to the genetic hyperlipidemia-associated pro-inflammatory / pro-atherogenic status in APOE knockout mice. To validate this hypothesis, in the current study we determined the impact of removal of the glucocorticoid function in APOE knockout mice on the outcome of two inflammation-associated pathologies, endotoxemia and atherosclerosis. Our studies show that adrenalectomy induces leukocytosis and enhances the susceptibility for endotoxemia in APOE knockout mice. The adrenalectomy-associated rise in white blood cells, however, does not alter atherosclerotic lesion development probably due to the parallel decrease in plasma levels of pro-atherogenic lipoproteins.

## Materials and Methods

### Mice

Homozygous APOE knockout mice were obtained from The Jackson Laboratory, crossed back to the C57BL/6 background (>8 generations) and bred in house at the Gorlaeus Laboratories, Leiden, The Netherlands. Since (1) female mice display both a higher basal corticosterone level [12,13] and a faster initial development of atherosclerotic lesions as compared to male mice under chow diet feeding conditions [14] and (2) the response of the hypothalamus-pituitary-adrenal axis is independent of the estrous cycle in mice [15], we used female APOE knockout mice that were fed a regular low-fat chow diet in all our experiments.

Mice were bilaterally adrenalectomized under isoflurane inhalation anesthesia through a dorsal midline skin incision and lateral retroperitoneal incisions. All efforts were made to minimize suffering. Parallel control (SHAM) operations were executed where the adrenals were touched but otherwise left intact. After closure of skin wounds using michel suture clip, operated mice were left separated from each other overnight for efficient wound healing and were subsequently housed with 4 similarly operated mice per cage. During the complete study, all mice were given 0.9% NaCl and normal water ad libitum and were regularly and identically handled. At the end of the study, no signs of endogenous adrenal regeneration were macroscopically visible in any of the adrenalectomized mice.

Animal experiments were performed in a temperature and light cycle (12h light/12h dark) controlled room at the Gorlaeus Laboratories of the Leiden/Amsterdam Center for Drug Research in accordance with the National Laws. All experimental protocols were approved by the Ethics Committee for Animal Experiments of Leiden University (Ethics protocol #10095).

### Corticosterone measurements

Blood samples for basal corticosterone analysis were drawn through tail chop between 9:00 and 10:00 AM (2- to 3h in the light period). Levels of corticosterone were determined using a ^125^I radio immuno assay (RIA) with a lower detection limit of 5 ng/ml, according to the manufacturer’s specifications (MP Biomedials). During blood draws mice were restrained for a maximum of 30 seconds.

### Blood cell analysis

At sacrifice (8 weeks post surgery) total white blood cells counts and the distribution over different subclasses of white blood cells were routinely measured using an automated SYSMEX XT-2000iV Veterinary Heamatology analyzer (SYSMEX Corporation). Verification of effects on specified white blood cell subclasses was performed using fluorescence activated cell sorting (FACS analysis) by staining cells with appropriate antibodies (CD11b, Ly6G, CD4, CD8, CD19, CD86, CD62L, all obtained form eBioscience, Belgium). For this purpose, blood was lysed using 0.83% NH_4_CL in 0.01 M Tris/HCL pH 7.2. Single-cell suspensions from spleen and thymus were obtained by squeezing the organs through a 70-µm cell strainer. Subsequently 300.000 cells were stained with the indicated antibodies. FACS analysis was performed on the FACSCalibur (Becton Dickinson, Mountain View, CA). Identification of specific leukocyte subsets was primarily based upon the indicated fluorescent antibody signals, but was further validated by checking the size and granularity of the tagged cells in the forward/side scatter plots. Data were analyzed using Cell Quest software.

### Lipopolysaccharide challenge

Mice were intravenously injected at 0900h with 50 μg/kg lipopolysaccharide (LPS) from Salmonella minnesota R595 (List Biological Laboratories Inc, Hornby, Canada) into the tail vein. Blood samples were collected after 30, 60, 90, and 120 minutes for cytokine analysis.

### Cytokine analyses

TNF-alpha and IL-6 protein levels in plasma were determined by ELISA (OptEIA kit, BD Biosciences Pharmingen, San Diego, CA) according to the suppliers protocols.

### Aortic root atherosclerotic lesion analysis

The arterial tree was perfused in situ with PBS (with the pressure of 100 mm Hg) for 10 minutes via a cannula in the left ventricular apex. The heart plus aortic root was excised and stored in 3.7% neutral-buffered formalin (Formalfixx ®, Shandon Scientific Ltd., UK). The atherosclerotic lesion areas in Oil red O stained cryostat sections of the aortic root were quantified using the Leica image analysis system, consisting of a Leica DMRE microscope coupled to a video camera and Leica Qwin Imaging software (Leica Ltd., Cambridge, UK). Mean lesion area (μm^2^) was calculated from >5 Oil red O-stained sections, starting at the appearance of the tricuspid valves. Lesion collagen content was determined using Masson’s Trichrome staining. All quantifications were done blinded by computer aided morphometric analysis using the Leica image analysis system.

### Plasma lipids

To determine the postprandial triglyceride response, mice were bled at 900h via the tail for basal plasma lipid values and subsequently given an oral dose of 200 μl olive oil. Blood samples were collected after 1, 2, 3, and 4 hours and plasma concentrations of total cholesterol and triglycerides were determined using enzymatic colorimetric assays (Roche Diagnostics). The basal lipoprotein distribution profile was analyzed by fractionation of 30 μl pooled plasma using a Superose 6 column (3.2 × 300 mm, Smart-system, Pharmacia). Total cholesterol, content of the effluent was determined using enzymatic colorimetric assays (Roche Diagnostics).

### VLDL triglyceride production

At 9:00 AM were injected intravenously with 500 mg of Triton WR1339 (Sigma) per kg body weight as a 15 g/dl solution in 0.9% NaCl to block plasma VLDL clearance. Blood samples were taken at 0, 1, 2, 3, and 4 hours after Triton WR1339 injection. Plasma triglycerides were analyzed enzymatically as described above. The hepatic VLDL production rate was calculated from the slope of the curve and expressed as mg/ml/h.

### Analysis of gene expression by real-time quantitative PCR

Quantitative gene expression analysis was performed as described [16]. Total RNA was isolated according to Chomczynski and Sacchi [17] and reverse transcribed using RevertAid^TM^ reverse transcriptase. Gene expression analysis was performed using real-time SYBR Green technology (Eurogentec). Primer sequences can be provided on request. Beta-actin (ACTB) and acidic ribosomal phosphoprotein P0 (36B4) were used as the standard housekeeping genes. Relative gene expression numbers were calculated by subtracting the threshold cycle number (Ct) of the target gene from the average Ct of beta-actin and 36B4 (Ct housekeeping) and raising 2 to the power of this difference. Genes that exhibited a Ct value of >35 were considered not detectable. The average Ct of two housekeeping genes was used to exclude that changes in the relative expression were caused by variations in the expression of the separate housekeeping genes.

### Data Analysis

Data are presented as means±SEM. Statistical analysis was performed using Graphpad Instat software (San Diego, USA, http://www.graphpad.com). Normality testing of the experimental groups was performed using the method Kolmogorov and Smirnov (Graphpad Instat). The significance of differences was calculated using a two-tailed Student’s t test or two way analysis of variance (ANOVA) with Bonferroni post-test where appropriate. Probability values less than 0.05 were considered significant.

## Results

**Figure 1:**
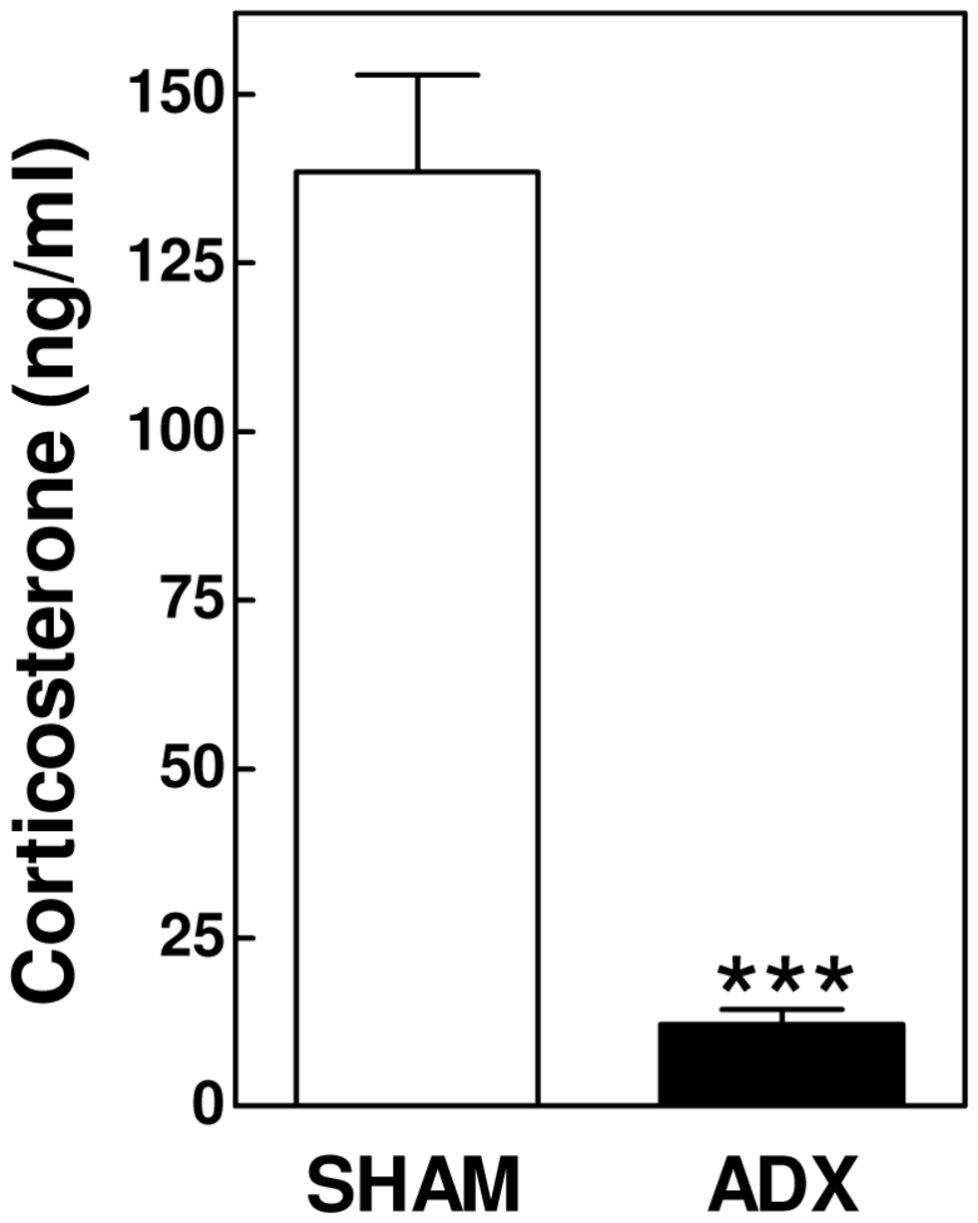
Adrenalectomized (ADX; N=19) APOE knockout mice show diminished plasma corticosterone levels as compared to SHAM-operated mice (N=22). Values represent means+SEM. *** P<0.001.

APOE knockout mice were subjected to bilateral adrenalectomy (ADX) to diminish their glucocorticoid function. In line with an essential role for the adrenals in the secretion of glucocorticoids, adrenalectomized APOE knockout mice exhibited markedly decreased plasma levels of corticosterone - the primary glucocorticoid circulating in mice - as compared to SHAM-operated control mice under basal conditions (12 ± 2 ng/ml vs 139 ± 14 ng/ml: P<0.001; Figure 1).

Routine blood analysis using a SYSMEX hematological analyzer revealed no major changes in red blood cell and platelet numbers and associated parameters (Table 1). In contrast, a marked increase in the white blood cell count (WBC; +55%; P<0.001) was noted after adrenalectomy (Table 1). This increase was primarily seen in the lymphocyte (+56%; P<0.001) and, to a minor extent, monocyte (+25%; P<0.05) population. Absolute neutrophil numbers were not changed in response to adrenalectomy, resulting in an overall increase in the lymphocyte white blood cell percentage (85 ± 1% vs 80 ± 1%; P<0.01) and a parallel decrease in the neutrophil (9.3 ± 0.8% vs 12.3 ± 0.9%; P<0.05) and monocyte fractions (4.2 ± 0.3% vs 5.4 ± 0.4%; P<0.05).

**Table 1:** Comparison of hematological parameters in the experimental groups.

Parameter	SHAM (N=12)	ADX (N=11)	P-value
Red blood cells (10E12/L)	10.0 ± 0.1	10.2 ± 0.2	> 0.05
MCV (fL)	49.5 ± 0.4	48.9 ± 0.2	> 0.05
HCT (%)	49.7 ± 0.4	49.9 ± 0.7	> 0.05
RDW-SD (fL)	30.8 ± 0.2	32.2 ± 0.3	**< 0.01**
Platelets (10E9/L)	1002 ± 63	988 ± 56	> 0.05
MPV (fL)	6.73 ± 0.04	6.73 ± 0.03	> 0.05
PCT (%)	0.68 ± 0.04	0.66 ± 0.04	> 0.05
PDW (fL)	7.50 ± 0.04	7.55 ± 0.07	> 0.05
White blood cells (10E9/L)	6.5 ± 0.5	10.0 ± 0.4	**< 0.001**
Neutrophils (10E9/L)	0.83 ± 0.10	1.11 ± 0.16	> 0.05
Lymphocytes (10E9/L)	5.4 ± 0.4	8.4 ± 0.4	**< 0.001**
Monocytes (10E9/L)	0.35 ± 0.03	0.44 ± 0.02	**< 0.05**
Eosinophils (10E9/L)	0.14 ± 0.02	0.17 ± 0.01	> 0.05
Neutrophils (%)	12.3 ± 0.9	9.3 ± 0.8	**< 0.05**
Lymphocytes (%)	80.2 ± 1.0	84.5 ± 0.7	**< 0.01**
Monocytes (%)	5.4 ± 0.5	4.2 ± 0.3	**< 0.05**
Eosinophils (%)	2.0 ± 0.2	1.6 ± 0.1	**< 0.05**

**Figure 2:**
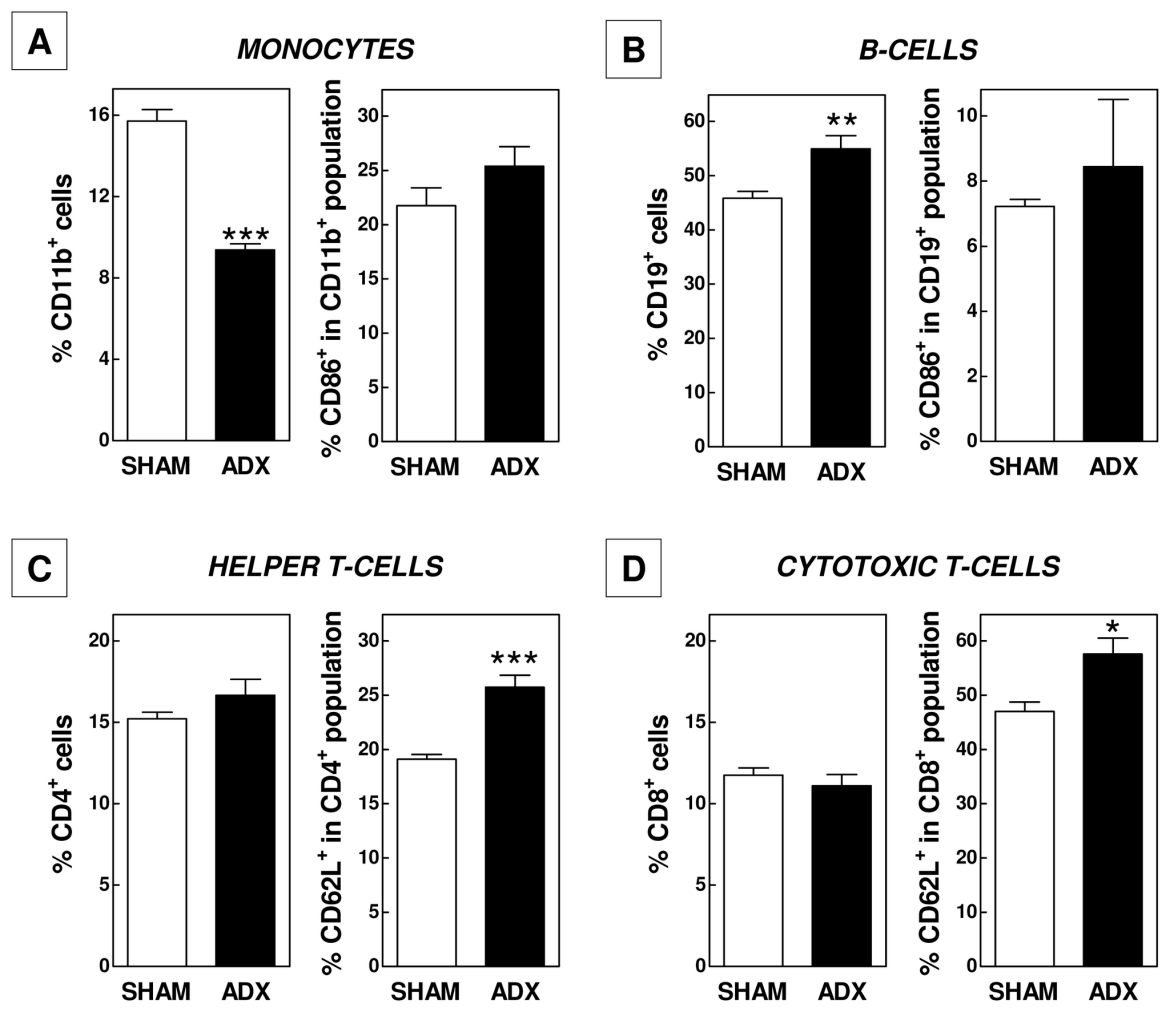
Adrenalectomy in APOE knockout mice decreases blood relative monocyte counts (A), increases B-lymphocyte numbers (B), and keeps T-helper cells (C) and cytotoxic T-cells (D) in a more immature state. Values represent means+SEM of 5 mice per group. *P<0.05, ** P<0.01, *** P<0.001.

To further specify the changes in leukocyte homeostasis we performed fluorescence activated cell sorting (FACS) analysis. In accordance with a decrease in the percentage of monocytes and neutrophils, the level of CD11b-positive cells as determined by FACS was significantly lower (9.4 ± 0.3% vs 15.7 ± 0.6%; P<0.001) in blood of adrenalectomized mice (Figure 2A). The activation state of the CD11b-positive cells - as measured by the presence of the surface marker CD86 – did not differ between groups (Figure 2A). Importantly, although the difference did not reach statistical significance due to the large intra-group variance (P>0.05), basal plasma levels of interleukin-6 were 51% higher in adrenalectomized mice (217±43 pg/ml; N=11) as compared to SHAM-operated controls (144±37 pg/ml; N=12). This indicates that cytokine secretion by macrophages was probably increased, which concurs with a more pro-inflammatory immune state in adrenalectomized mice.

**Figure 3:**
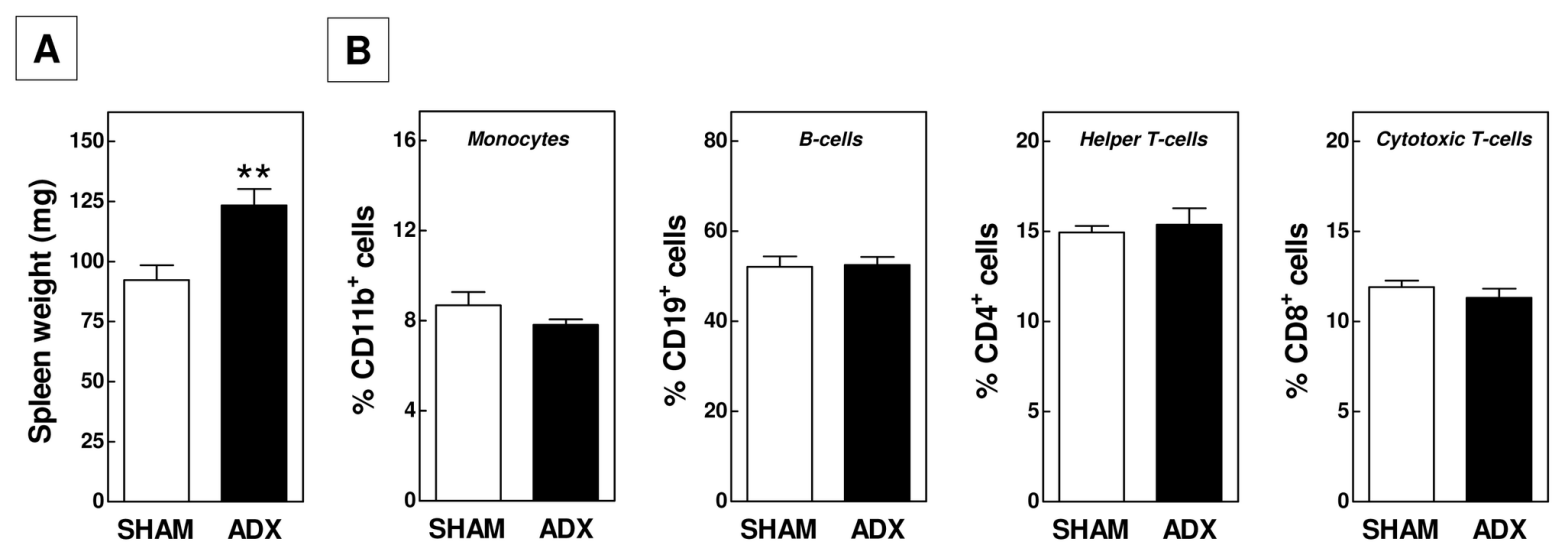
Absolute spleen weights (A) and relative amounts of monocytes, B- and T-lymphocyte populations in spleens (B) of adrenalectomized (ADX) and SHAM-operated APOE knockout mice. Values represent means+SEM of 5 (FACS data) or 10-15 (spleen weight) mice per group. ** P<0.01.

Despite an identical expression level of CD86 on B-lymphocytes, the blood of adrenalectomized mice was significantly enriched in CD19-positive cells (55 ± 2% in ADX vs 46 ± 1% in SHAM; P<0.01; Figure 2B). The spleen serves as an important reservoir of B-cells. In further support of a generally higher systemic inflammatory status due to removal of the glucocorticoid function, spleen weight increased from 92 ± 6 mg in SHAM-operated mice to 123 ± 7 mg in adrenalectomized mice (P<0.01; Figure 3). However, FACS analysis did not reveal differences in the relative number (Figure 3) or activation state (data not shown) of splenic CD11b-positive cells and B- and T-lymphocyte populations.

**Figure 4:**
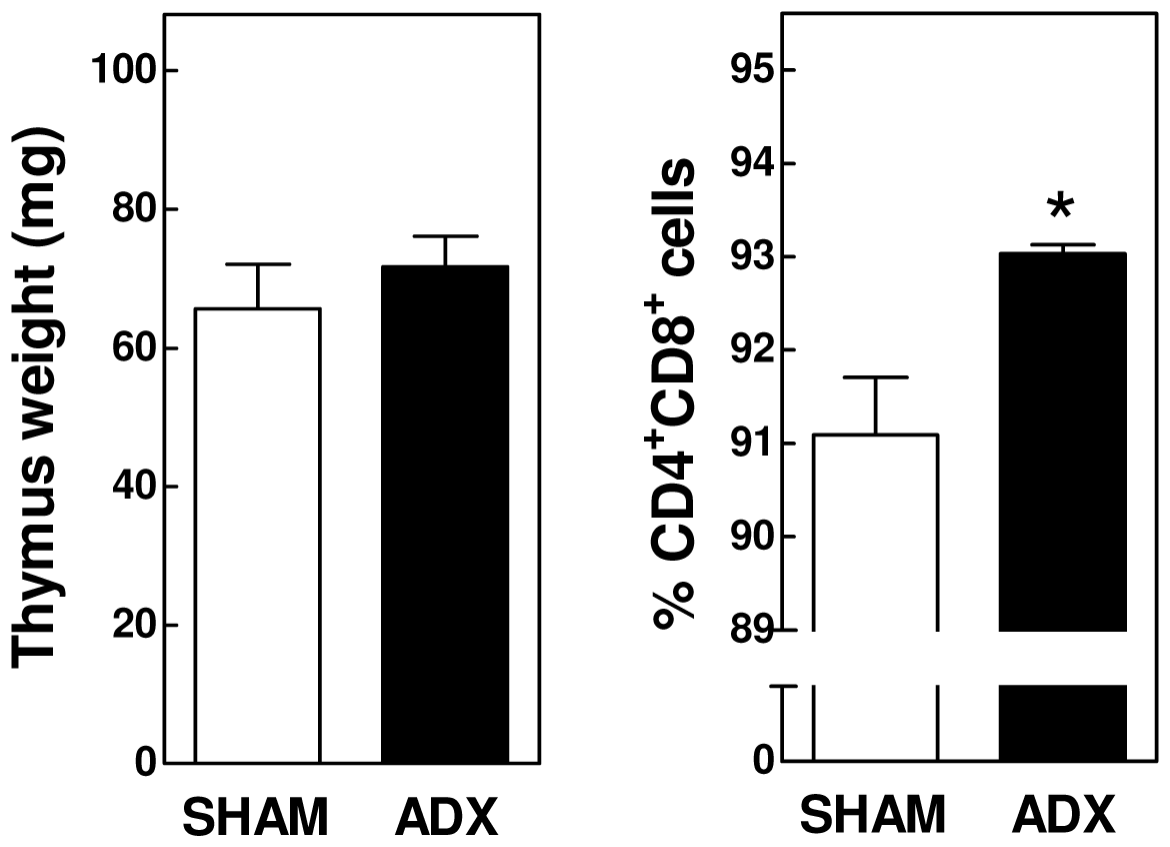
Adrenalectomy in APOE knockout mice does not alter the absolute thymus weight, but increases the thymic CD4/CD8 double positive lymphocyte fraction. Values represent means+SEM of 5 (FACS data) or 10-15 (thymus weight) mice per group. * P<0.05.

In both groups of mice, CD4-positive T-helper cells (Figure 2C) and CD8-positive cytotoxic T-lymphocytes (Figure 2D) constituted 11-16% of total blood leukocytes. We detected a significant change in the T-lymphocyte maturity level upon adrenalectomy. The fraction of cells expressing the immaturity marker CD62L was respectively 34% (P<0.001) and 22% (P<0.05) higher for CD4-positive and CD8-positive cells in the ADX group as compared to the SHAM-operated group in blood (Figure 2C/2D). Taking the 1.5-fold increase in total white blood cell numbers into account, it thus appears that adrenalectomized mice show a marked increase in the absolute number of immature (+102% for CD4; +84% for CD8) but not in mature T-lymphocytes. Thymus weight was not significantly different in the two groups of mice (Figure 4). However, a marginal but significant increase in the fraction of CD4/CD8 double positive thymocytes could be observed in ADX mice (93.0 ± 0.2% vs 91.1 ± 1.4%; P<0.05; Figure 4). Together, these findings suggest a stimulated development in and secretion of naïve T-cells from the thymus upon removal of the glucocorticoid function by adrenalectomy.

**Figure 5:**
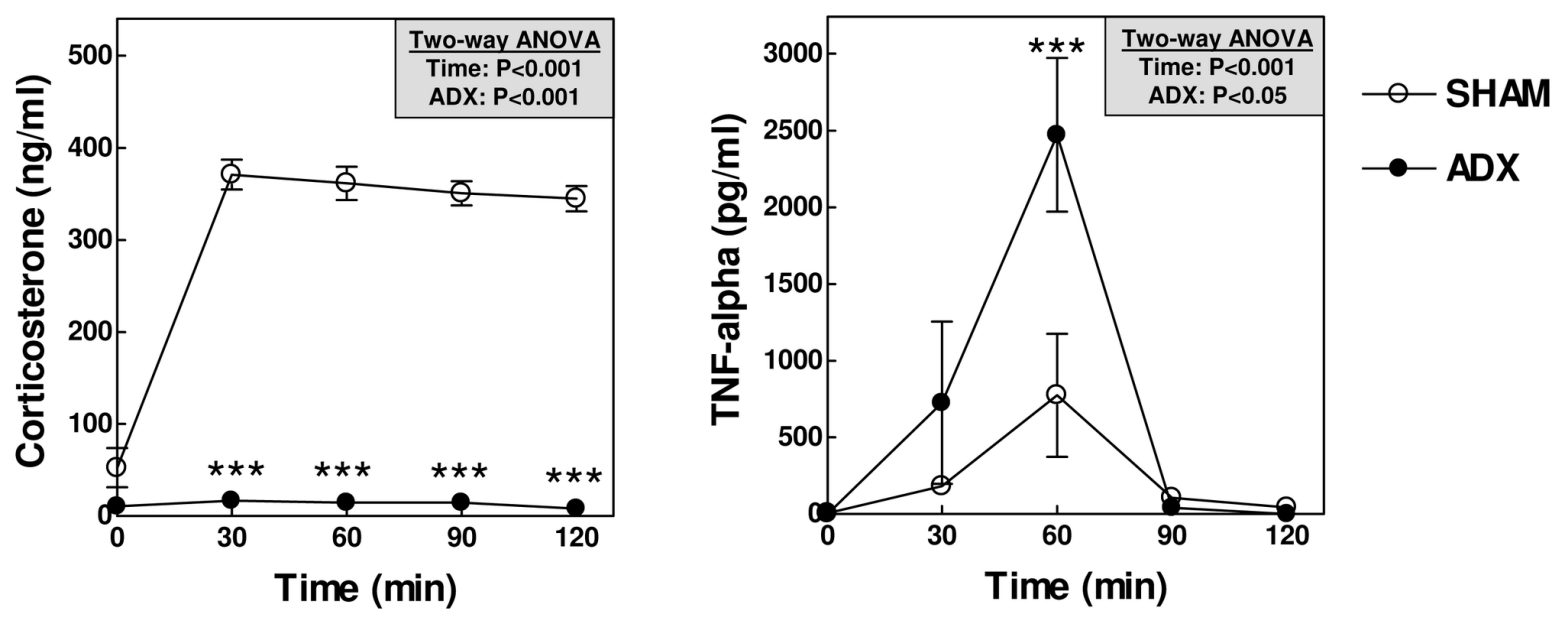
Adrenalectomized APOE knockout mice fail to increase their plasma corticosterone levels upon exposure to a sub-lethal dose of LPS, which is associated with a markedly enhanced plasma TNF-alpha response. Values represent means+SEM of 5 (SHAM) and 4 (ADX) mice per group. *** P<0.001.

To analyze the impact of adrenalectomy on the sepsis susceptibility adrenalectomized mice and SHAM controls were subjected to a sub-lethal dose of lipopolysaccharide (LPS; 50 μg/kg) to stimulate secretion of the pro-inflammatory cytokine tumor necrosis factor-alpha (TNF-alpha) by macrophages. As depicted in Figure 5, only SHAM-operated mice were able to increase the plasma level of corticosterone in response to the LPS-induced pro-inflammatory trigger. TNF-alpha levels were not detected in plasma of adrenalectomized or SHAM-operated mice that had not been subjected to a pro-inflammatory trigger. As expected, LPS exposure induced a time-dependent increase in plasma levels of TNF-alpha in SHAM-operated mice. Importantly, adrenalectomy markedly stimulated the LPS-induced TNF-alpha response (Figure 5). This was paralleled by such severe disease symptoms (immobility; hypothermia) that the adrenalectomized mice had to be euthanized early, i.e. after 2.5 hours into the experiment. It thus seems that in APOE knockout mice glucocorticoids do efficiently execute their anti-inflammatory function and thereby protect against induction of (fatal) endotoxemia.

**Figure 6:**
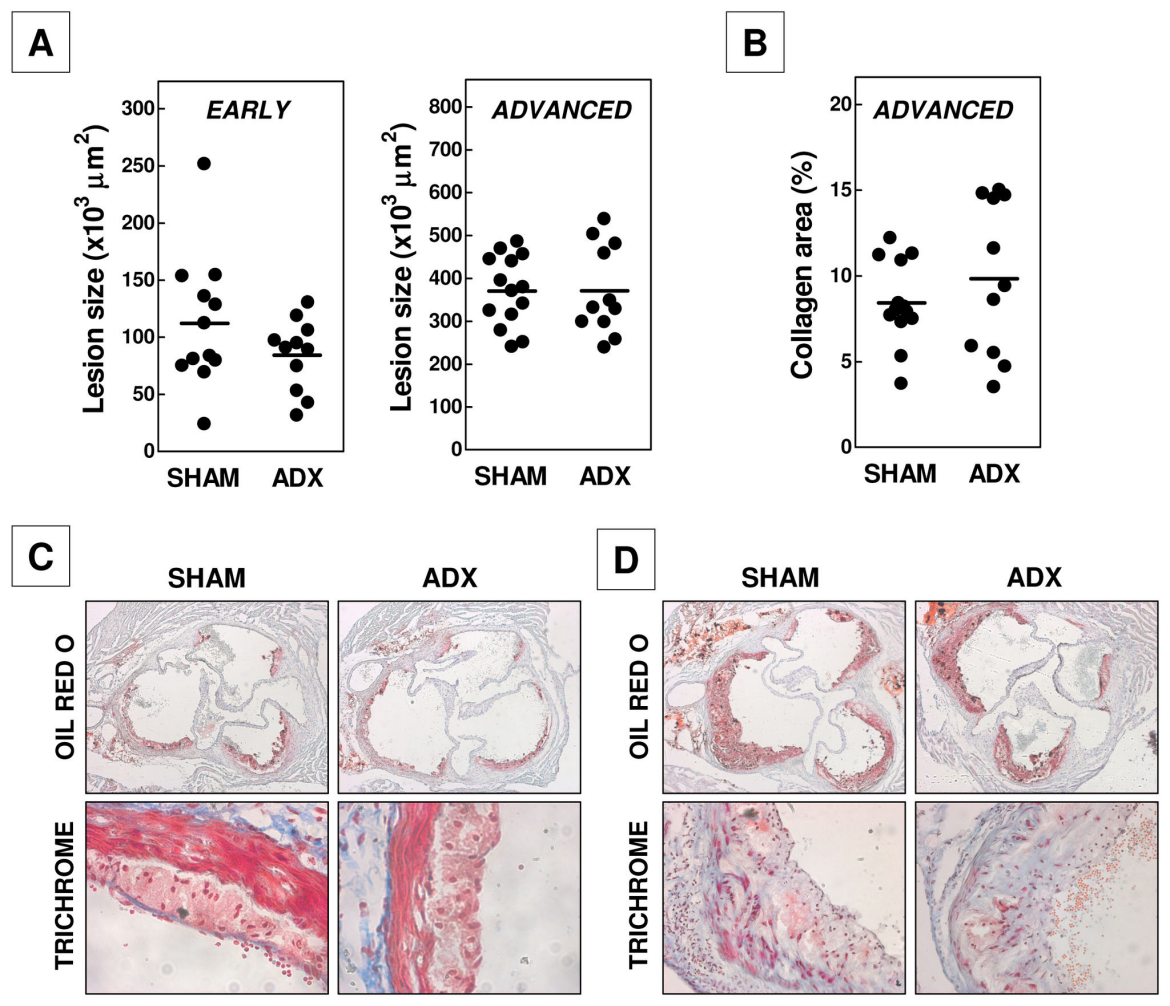
Adrenalectomy does not alter the size (A) or collagen content (B) of atherosclerotic lesions in APOE knockout mice. Representative neutral lipid (Oil red O) and collagen (Trichrome: blue) stainings of early (C) and advanced (D) aortic root lesions. Separate dots in figures A and B represent values for each individual mouse, while horizontal lines indicate the respective group averages.

Development of atherosclerotic lesions is a slow process that progresses over a period of weeks or months depending on the type of genetically modified mice and diet used. To study the impact of the glucocorticoid function on the initial phase of atherosclerotic lesion development in our APOE knockout mice, we performed ADX or SHAM operations in age-matched 5- to 6-week old mice and subsequently waited for 8 weeks for lesions to develop while feeding the mice a regular chow low fat diet without added cholesterol. As evident from Figure 6A, adrenalectomy did not increase the susceptibility for the development of initial atherosclerotic lesions. Both groups of operated mice exhibited a similar extent of atherosclerosis development at the aortic root with respective lesion sizes of 112 ± 17 x 10^3^ μm^2^ for SHAM-operated mice and 84 ± 9 x 10^3^ μm^2^ for ADX mice. In line with the macrophage-rich fatty streak lesion stage, virtually no collagen was detected in any of the lesions in either experimental group (<1%; data not shown). Since the effect of interventions in the atherogenic process may dependent on the stage of lesion development, we also examined the impact of glucocorticoid depletion on the progression of atherosclerotic lesions from the initial macrophage-rich stage to more advanced collagen-containing plaques. Hereto, we executed the adrenalectomy in age-matched 14 weeks old mice and quantified the lesion content at 22 weeks of age. Similarly as observed for the initial development of atherosclerotic lesions, progression of initial lesions to more advanced atherosclerotic plaques was unaffected by adrenalectomy (Figure 6B). Both groups of operated mice displayed aortic root lesions of about 370 x 10^3^ μm^2^ that contained significant amounts of collagen (8-9% of total lesion area). In contrast to the increased sepsis severity noted after adrenalectomy, the higher inflammatory status associated with removal of the glucocorticoid function thus did not translate into a stimulated susceptibility for atherosclerotic lesion development.

**Figure 7:**
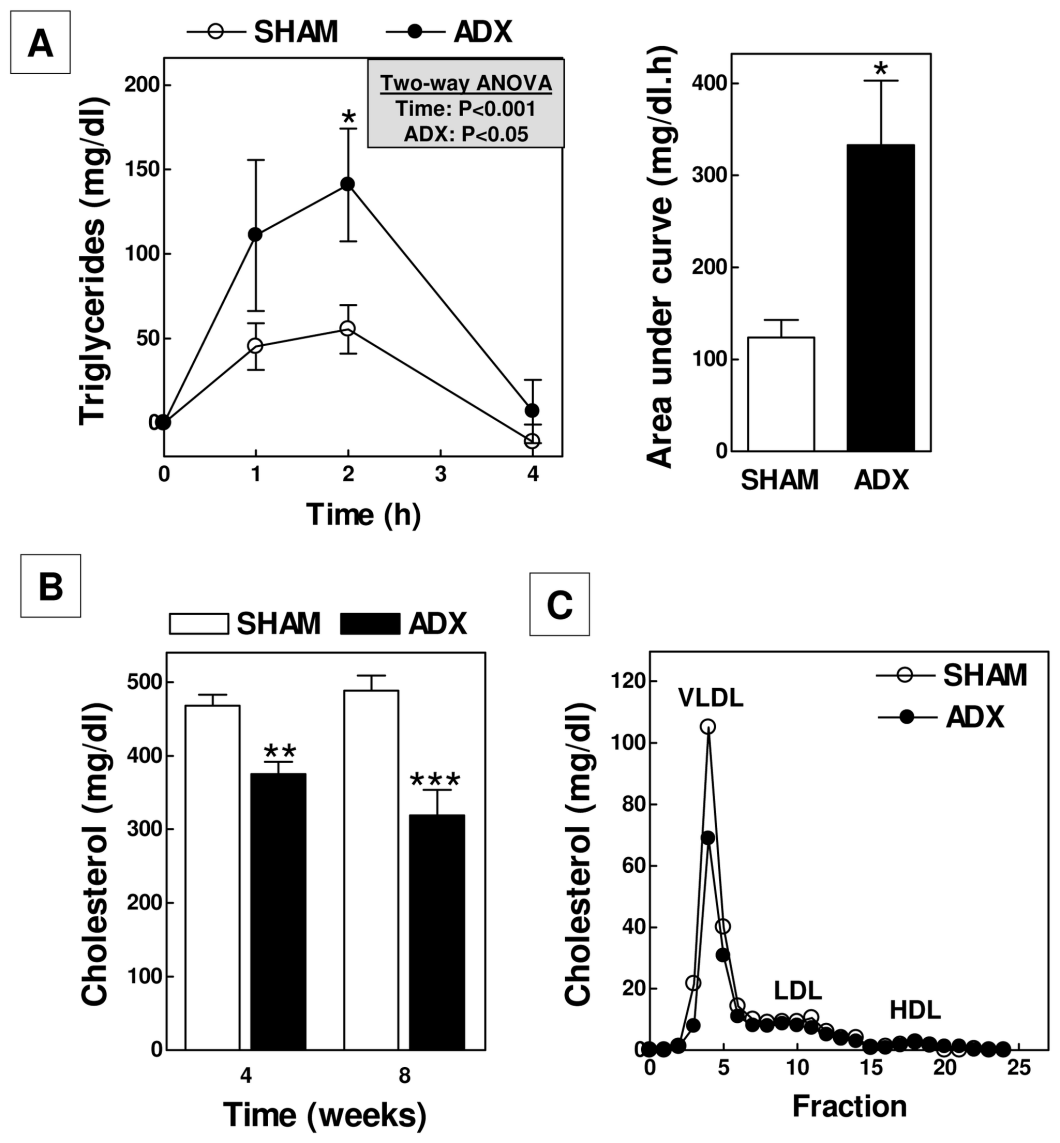
Plasma postprandial triglyceride response (A), cholesterol levels (B), and lipoprotein profiles (C) in adrenalectomized (ADX) and SHAM-operated APOE knockout mice. VLDL, very low-density lipoprotein; LDL, low-denisty lipoprotein; HDL, high-density lipoprotein. Values represent means+SEM or 5 mice per group. * P<0.05, ** P<0.01, *** P<0.001.

**Figure 8:**
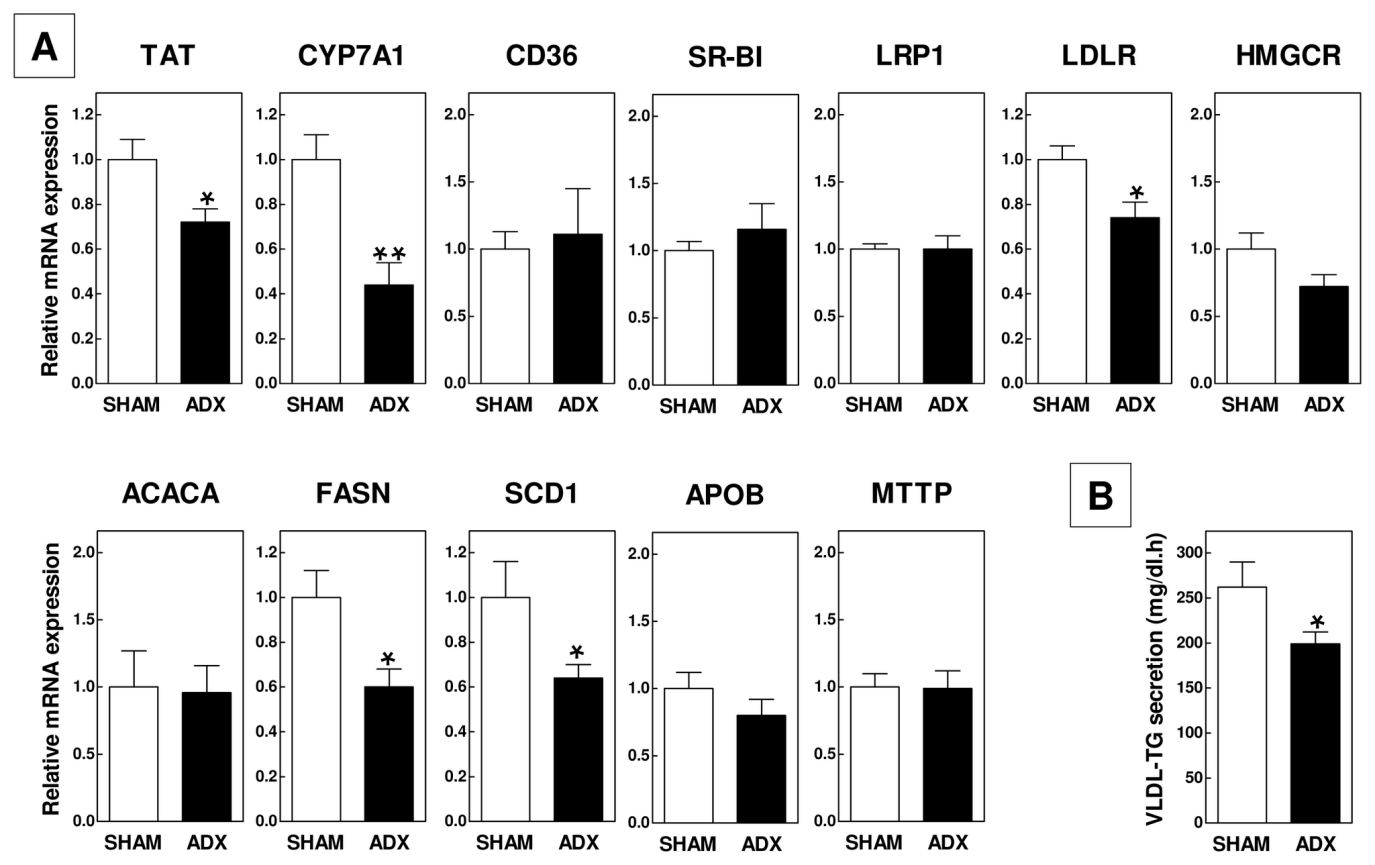
Relative gene expression levels in liver (A) and hepatic VLDL-triglyceride secretion rates (B) of adrenalectomized (ADX) and SHAM-operated APOE knockout mice. Values represent means+SEM of 5-8 mice per group. * P<0.05, ** P<0.01.

Recent studies have suggested a new role for endogenous glucocorticoids in the suppression of intestinal cholesterol absorption [18]. We measured the postprandial triglyceride response upon an oral fat load to determine whether glucocorticoids also inhibit intestinal triglyceride mobilization. Ad libitum-fed basal plasma triglyceride levels were low and not different between the two groups of mice (98±13 mg/dl for ADX (n=10) vs 99±7 mg/dl for SHAM (n=15); P>0.05). Administration of a bolus of olive oil induced a time-dependent increase in plasma triglyceride levels in SHAM-operated mice, which peaked at 2 hours and returned to basal levels 4 hours after the treatment (Figure 7A). The peak of the increase in triglyceride levels and the area-under-the-curve of the total postprandial response were respectively 2.5-fold and 2.7-fold higher in ADX mice (P<0.05 for both; Figure 7A). In line with the notion that the increase in the postprandial triglyceride response was due to increased intestinal lipid absorption, we did not detect a change in the hepatic expression level of the key lipolytic enzyme lipoprotein lipase (LPL; data not shown). Strikingly, plasma total cholesterol levels were decreased in adrenalectomized mice (Two-way ANOVA P<0.001 for ADX; Figure 7B), with a 20% decrease (P<0.01) four weeks post surgery and a marked 35% decrease (P<0.001) at sacrifice 8 weeks post ADX. Lipoprotein distribution analysis revealed that the decrease in total cholesterol noted after 4 weeks could be primarily attributed to a decrease in the level of very-low-density lipoproteins (VLDL; -34%) – the main lipoprotein species circulating in blood of APOE knockout mice – as plasma levels of low-density lipoproteins (LDL; -18%) and high-density lipoproteins (HDL; +6%) were much less affected (Figure 7C). Several processes play a crucial role in the control of steady state plasma VLDL-cholesterol levels, including secretion of triglyceride-rich VLDL particles by the liver and removal of VLDL-remnants and LDL from the blood circulation through receptor-mediated uptake by hepatocytes. To uncover the mechanism behind the decrease in VLDL levels observed in ADX mice, the effect of adrenalectomy on hepatic gene expression levels was determined (Figure 8A). In accordance with a diminished glucocorticoid signalling in the liver, we noted a significant decrease in the liver mRNA expression of glucocorticoid receptor targets tyrosine aminotransferase (TAT; -28%; P<0.05) and cholesterol 7alpha-hydroxylase (CYP7A1; -56%; P<0.01). Relative mRNA expression levels of the lipoprotein receptors CD36, scavenger receptor BI (SR-BI) and LDL receptor related protein 1 (LRP1) were not different between groups. LDL receptor (LDLR) transcript levels were significantly lower in ADX mice (-26%; P<0.05), suggesting a diminished hepatic clearance of specifically apolipoprotein B100 (APOB100)-containing lipoproteins. The expression levels of the rate-limiting enzyme in de novo synthesis of cholesterol from acetyl-CoA, HMG-CoA reductase (HMGCR), were not significantly changed. The first committed step of fatty acid synthesis – the conversion of acetyl-CoA to malonyl-CoA – did also not seem to be affected as judged from the unaltered expression of acetyl-CoA carboxylase (ACACA). Adrenalectomy was, however, associated with a respective 36% and 40% (P<0.05 both) decrease in the expression of fatty acid synthase (FASN) and stearoyl-Coenzyme A desaturase 1 (SCD1), lipogenic enzymes that carry out essential steps in the synthesis of fatty acids from acetyl-CoA and malonyl-CoA. Microsomal triglyceride transfer protein (MTP) and APOB expression levels were not different between ADX and SHAM mice, indicative of a similar capacity of the liver to package triglycerides into APOB-containing VLDL particles. Importantly, the changes in lipogenic gene expression did translate into an overall 24% lower (P<0.05; Figure 8B) hepatic secretion rate of triglyceride-rich VLDL particles.

## Discussion

Glucocorticoids are considered to be some of the most powerful endogenous anti-inflammatory molecules. Both natural and synthetic glucocorticoids have therefore emerged as principal therapeutic agents to treat inflammation-based diseases, such as rheumatoid arthritis (RA) [19] and systemic lupus erythematosus (SLE) [20]. In the current study, the impact of removal of the glucocorticoid function through adrenalectomy on the basal inflammatory status, sepsis susceptibility, and atherosclerotic lesion development was evaluated in hyperlipidemic APOE knockout mice. Although we anticipate that the majority of the effects induced by adrenalectomy are secondary to disruption of the glucocorticoid function, it should be noted that ablation of the adrenal catecholamine and mineralocorticoid function may also have contributed to the overall outcome of our studies.

In accordance with suppression of the proliferation and maturation of pro-inflammatory cell types by glucocorticoids, we observed a rise in the blood leukocyte count and an increase in spleen weight, which could be primarily attributed to a higher number of B- and T-lymphocytes and a minor increase in monocytes. Previous findings from adrenal insufficient melanocortin receptor 2 knockout mice have already suggested an important role for endogenous glucocorticoid in B-cell development. Melanocortin receptor 2 knockout mice display an increase in all splenic B-cell fractions, which coincided with a higher number of pre-B cells in the bone marrow [21]. However, a diminished apoptosis rate of mature B cells [22] may also underlie the increase in B-cell numbers observed upon adrenalectomy. Based upon our FACS analysis it seems that the increase in blood T-lymphocyte numbers may originate from a stimulated production of T-lymphocytes in the thymus as we detected an increase in the CD4/CD8 double positive thymocyte fraction upon adrenalectomy. These findings concur with data from Purton et al. on the high sensitivity of double positive thymocytes for glucocorticoid-induced death [23] and previous observations of increased double positive thymocyte numbers in adrenalectomized rats [24] and melanocortin receptor 2 knockout mice [21].

APOE knockout mice as compared to normolipidemic wild-type mice display an enhanced susceptibility to endotoxemia [6,7]. Our present study suggests that the enhanced glucocorticoid levels in APOE knockout mice protect against (lethal) sepsis, since adrenalectomy in APOE knockout mice was associated with a concomitant higher susceptibility for septic shock as judged from the increased TNF-alpha response and more extreme disease symptoms / lethality upon LPS exposure. Monocyte lineages play an essential role in the pathogenesis of endotoxemia as monocyte-derived macrophages secrete pro-inflammatory cytokines that - when their levels reach an upper threshold level - induce septic shock. The higher endotoxemia susceptibility may therefore in part directly result from the monocytosis observed upon adrenal removal. However, glucocorticoids can also diminish the secretion of pro-inflammatory cytokines by leukocytes and endothelial cells through inhibition of NFkappaB action [25], which may also explain the increase in plasma TNF-alpha levels in ADX mice. Furthermore, our data clearly indicate that, although APOE may exhibit potent anti-inflammatory properties, its presence is not essential for the protection of glucocorticoids against inflammation.

Relatively high numbers of blood leukocytes are significant contributors to cardiovascular disease - i.e. coronary artery disease and ischemic heart disease - in the human situation [26-30]. In striking contrast to the marked increase in sepsis susceptibility, the leukocytosis observed upon adrenalectomy however did not affect either the initiation or progression of atherosclerotic lesions in our APOE knockout mice. Our data further highlight the complexity regarding the effect of glucocorticoids on atherosclerotic lesion development and cardiovascular disease incidence. In the human situation, low glucocorticoid levels as observed in Addison’s disease (primary adrenal insufficiency) are associated with a higher cardiovascular mortality rate as well as an increased rate of death due to infectious diseases [31], which is probably related to the diminished glucocorticoid-mediated anti-inflammatory action. In parallel, adrenalectomy stimulated atherosclerotic lesion development in atherosclerosis-susceptible LDL receptor knockout mice fed a pro-inflammatory cholic acid-containing atherogenic diet [9]. In contrast, disruption of glucocorticoid receptor function in macrophages alters the intra-plaque calcification rate but does not affect atherosclerotic lesion size in Western-type diet-fed LDL receptor knockout mice [32]. Patients suffering from Cushing’s syndrome – hypercortisolemia – do actually also exhibit an enhanced risk for cardiovascular disease [33]. This has been attributed to the pro-atherogenic metabolic phenotype (i.e. diabetes, hypertension) associated with long-term high plasma glucocorticoid levels. Lowering of plasma pro-atherogenic lipid levels by statin treatment is the primary intervention to effectively reduce the cardiovascular disease risk in human subjects [34]. Importantly, in the current study we detected a significant decrease in VLDL-cholesterol levels that could be explained by a diminished secretion of triglyceride-rich VLDL particles by the liver as a result of decreased hepatic lipogenesis. It is therefore anticipated that the absence of a pro-atherogenic outcome in response to the leukocytosis associated with adrenalectomy may be attributed to a parallel decrease in the pro-atherogenic lipid trigger. In accordance, disruption of specifically glucocorticoid’s metabolic function through administration of an 11β-hydroxysteroid dehydrogenase type 1 (11β-HSD1) inhibitor is able to improve the plasma lipoprotein profile and decrease atherosclerotic plaque load in APOE knockout mice [35] and agouti LDL receptor knockout mice [36]. From these combined findings it appears that a sensitive balance between anti-inflammatory / anti-atherogenic actions and pro-atherogenic metabolic consequences of glucocorticoids exists that determines the overall outcome of the atherosclerosis and cardiovascular disease. From a therapeutical point of view it thus seems of critical importance to take into account both the general metabolic and inflammatory status of human subjects upon treatment of glucocorticoid-related diseases or when administering glucocorticoid-based drugs, especially if one also considers the potent protective properties of glucocorticoids in the context of sepsis and other inflammatory pathologies.

In conclusion, our studies show that adrenalectomy induces leukocytosis and enhances the susceptibility for endotoxemia in APOE knockout mice. The adrenalectomy-associated rise in white blood cells, however, does not alter atherosclerotic lesion development probably due to the parallel decrease in plasma levels of pro-atherogenic lipoproteins.
